# Systematic Review of Anthocyanins and Markers of Cardiovascular Disease

**DOI:** 10.3390/nu8010032

**Published:** 2016-01-09

**Authors:** Taylor C. Wallace, Margaret Slavin, Cara L. Frankenfeld

**Affiliations:** 1 Department of Nutrition and Food Studies, George Mason University, Fairfax, VA 22030, USA; 2 Department of Global and Community Health, George Mason University, Fairfax, VA 22030, USA

**Keywords:** anthocyanins, cardiovascular disease, LDL cholesterol

## Abstract

Anthocyanins are dietary flavonoids commonly consumed in the diet, which have been suggested to have a preventative effect on cardiovascular disease (CVD) development among epidemiological studies. We systematically reviewed randomized controlled trials (RCTs) testing the effects of purified anthocyanins and anthocyanin-rich extracts on markers of CVD (triglycerides, total cholesterol, low-density lipoprotein (LDL) cholesterol, high-density lipoprotein (HDL) cholesterol, and blood pressure) in both healthy and diseased populations. Eligible studies included RCTs of adults published in English. We searched PubMed, Web of Science Core Collection, and BIOSIS Previews for relevant articles from inception until 1 July 2014. Twelve RCTs representing 10 studies were included in this review. Supplementation with anthocyanins significantly improved LDL cholesterol among diseased individuals or those with elevated biomarkers. Supplementation did not significantly affect other markers of CVD in either healthy individuals or those with elevated markers. No adverse effects of anthocyanins were reported across studies at levels up to 640 mg/day. Limitations of trials in the qualitative analyses include short trial duration and large variability in the dose administered within the trials. Longer-duration trials assessing dose response are needed to adequately determine whether an effect of supplementation exists.

## 1. Introduction

Cardiovascular disease (CVD) is the number one cause of death worldwide, according to the World Health Organization (WHO). The WHO predicts that by 2030, over 28 million individuals will die from CVD annually [1]. Over the past decade, there has been increased interest in lifestyle and dietary interventions to reduce CVD risk. Research has shown that individuals who adhere to US national guidelines for a healthful diet [2] and physical activity [3] have lower cardiovascular morbidity and mortality than those who do not adhere to these guidelines. Higher fruit and vegetable consumption has been suggested to be inversely associated with a decreased risk of CVD [2]. Berry consumption has recently been reviewed and has shown to be an essential fruit group in a heart-healthy diet [4]. This may be due in part to the abundance and variety of dietary bioactive components present in plant foods.

Anthocyanins are the red-orange to blue-violet pigments present in many fruits, vegetables, flowers, grains, and other plant-derived foods. Interest in the biological effects of anthocyanins has grown because of their noted presence in the human diet, as well as their potential use as a value-added alternative to synthetic colorants in many food products. Evidence from epidemiological studies supports potential preventative effects of these compounds toward the onset of CVD [5,6,7,8] in a dose-response manner in both men and women [6]. Animal and *in vitro* cell studies support biological plausibility for these compounds to favorably improve validated and surrogate biomarkers of CVD [9].

In humans, several small to medium-sized randomized controlled trials (RCTs) have assessed the effects of purified anthocyanins and anthocyanin-rich extracts on validated biomarkers of CVD in populations of both healthy and diseased adults (*i.e.*, those with elevated markers). A plethora of clinical evidence and expert reviews support increased consumption of anthocyanin-rich whole foods and CVD prevention [4]; however, to our knowledge, there is no systematic review that assesses the effect of purified anthocyanins and/or anthocyanin-rich extracts on markers of cardiovascular health among RCTs. The objective of this study was to systematically review these RCTs and to identify research gaps where additional scientific evidence is warranted. For this systematic review, we chose to evaluate validated and/or common markers used clinically as biomarkers of cardiovascular health and to diagnose cardiovascular diseases (*i.e.*, lipids, triglycerides and blood pressure). Many types of inflammatory markers have also been measured across clinical studies; however, we chose not to review these markers because of their limited clinical use and high inter- and intra-assay variability.

## 2. Materials and Methods

We conducted this systematic review according to the Cochrane and Preferred Reporting Items for Systematic Reviews and Meta-Analyses (PRISMA) guidelines [10,11]. This systematic review also takes into account the recommendations of Lichtenstein *et al.* [12] and Moher and Tricco [13], which highlight areas unique to the field of nutrition, that are important to consider throughout the systematic review process. 

### 2.1. Literature Search

We searched three databases (PubMed, Web of Science Core Collection, and BIOSIS Previews) for relevant articles from inception until 1 July 2014 using the following search algorithm: (anthocyanins AND (“cholesterol, hdl” OR “cholesterol, ldl” OR “cholesterol, vldl” OR triglycerides OR lipoproteins OR hypertension)). Supplementary literature searches included examining the reference lists of all relevant studies, pertinent review articles, and the Cochrane Library Database to identify articles not identified in our initial electronic search.

### 2.2. Study Selection

Studies were eligible for inclusion if the following applied: (1) they were RCTs that compared purified anthocyanins or anthocyanin-rich extracts against a placebo control; (2) they involved adult participants aged ≥18 years old; (3) they assessed the effect of purified anthocyanins or anthocyanin-rich extracts on markers of CVD (triglycerides, total cholesterol, high-density lipoprotein (HDL) cholesterol, low-density lipoprotein (LDL) cholesterol, or blood pressure); (4) the treatment group(s) reported a quantitative or quantifiable anthocyanin content; and (5) they were published in English. Three investigators (T.C.W., C.L.F., and M.S.) independently screened the titles and abstracts of articles for eligibility for inclusion in the systematic review. If consensus was reached, ineligible articles were excluded and eligible articles were moved to the next stage (full-text review) in the process. If consensus was not reached, the article was moved to the next stage, in which the full text of the selected articles was evaluated to determine the eligibility for inclusion in the systematic review. Disagreements were resolved by discussion among the reviewers until a consensus was reached.

**Figure 1:**
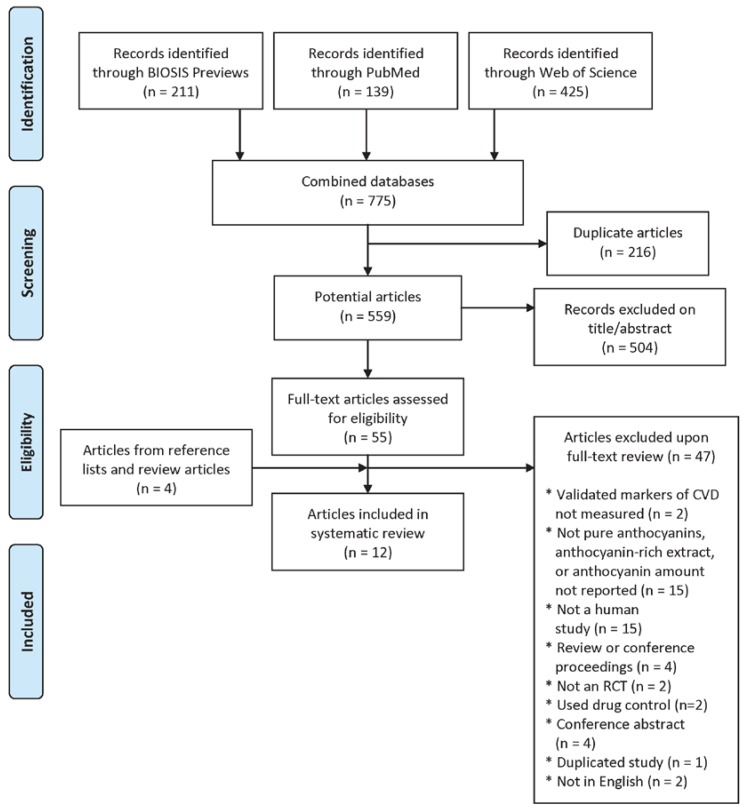
Flow diagram of article selection.

Based on the title and abstract review, 55 eligible articles were included in the full-text review. In addition to the studies identified in the literature search, four other articles were identified from the reference lists of full-text reviewed studies and were retrieved for full-text screening (59 total studies). Based on the full-text review, 47 articles were excluded, including two studies that did not assess validated markers of CVD, 15 studies that did not assess the effect of purified anthocyanins or anthocyanin-rich extracts and/or did not report the amount of anthocyanins in the treatment, 15 non-human studies, four review or conference proceedings manuscripts, two trials that were not randomized, two trials that did not use a placebo control, four conference abstracts, one duplicated study (data published twice in separate journals), and two studies not in English (Figure 1).

### 2.3. Data Synthesis

One investigator (C.L.F.) extracted key information from selected studies (Table 1) and two investigators (M.S. and T.C.W.) independently verified the extracted data for completeness and accuracy. The investigators resolved disagreements by consensus. Data extracted included country, sample size, participant characteristics (e.g., age, gender, and health status), dosage and duration of the treatment or intervention, follow-up period, primary end point(s), and main findings for CVD outcomes. Meta-analysis and/or meta-regression were not performed because the qualitative assessment of heterogeneity of the RCTs indicated that it would be inappropriate to statistically combine the studies. Assessment of heterogeneity included the duration of the trials (3–24 weeks), dose of anthocyanins administered (7.35–640 mg/day), raw material source of anthocyanins and therefore composition of individual anthocyanins, and the age, gender, health status, and body mass index of the studied populations.

**Table 1:** Study characteristics ^1^.

Reference	Country	Design	No. Randomized	No. Completed	Gender	Age (Year)	BMI or Weight Status	CVD-Related Disease Status	Intervention	Extract Dose	Anthocyanin Dose	Control	Intervention Length	End Point(s)
Curtis *et al.*, 2009 [14]	United Kingdom	Parallel	57	52	Postmenopausal women	<70	20–32	Healthy	Elderberry extract capsule	NR	500 mg/day	Placebo capsule	12 weeks	Triglycerides, TC, LDL, HDL, SBP, and DBP
Gurrola-Díaz *et al.*, 2010 [15]	Mexico	Parallel	152	124	Women and men	30–71	NR	Healthy and MetS	*Hibiscus sabdariffa* extract powder + preventative diet	100 mg/day	19.24 mg/day	Preventive diet	1 month	Triglycerides, TC, LDL, HDL, SBP, and DBP
Hansen *et al.*, 2005 [16]	Denmark	Parallel	70	69	Women and men	38–75	Mean 25	Healthy	Red grape extract	Full or half dose (unclear)	71 mg/day for men; 48 mg/day for women; 36 mg/day for men; 24 mg/day for women	Placebo capsule (microcrystalline cellulose)	4 weeks	TC, LDL, HDL, SBP, and DBP
Hassellund *et al.*, 2012 [17]	Norway	Crossover	31	27	Men	35–51	Mean 27	Prehypertensive	Purified anthocyanins from bilberry and black currant	NA	640 mg/day	Placebo capsule (maltodextrin)	4 weeks	SBP and DBP
Hassellund *et al.*, 2013 [18]	Norway	Crossover	31	27	Men	35–51	NR	Prehypertensive	Purified anthocyanins from bilberry and black currant	NA	640 mg/day	Placebo capsule (maltodextrin + blue color)	4 weeks	Triglycerides, TC, LDL, and HDL
Karlsen *et al.*, 2007 [19]	Norway	Parallel	120	118	Women and men	40–74	Mean 25	Healthy	Purified anthocyanins from bilberry and black currant	NA	300 mg/day	Placebo capsule (maltodextrin + blue color)	3 weeks	TC and HDL
Kianbakht *et al.*, 2014 [20]	Iran	Parallel	105	80	Women and men	20–60	Mean 30	Primary hyperlipidemia	Whortleberry extract	1050 mg/day	7.35 mg/day	Placebo capsule (toast powder)	2 months	Triglycerides, TC, LDL, and HDL
Naruszewicz *et al.*, 2007 [14]	Poland	Parallel	NR	44	Postmenopausal women, and men	Mean 66	Mean 26	Post-MI	Chokeberry extract	255 mg/day	63.75 mg/day	Placebo capsule (maltodextrin)	6 weeks	Triglycerides, TC, LDL, HDL, SBP, and DBP
Qin *et al.*, 2009 [21]	China	Parallel	NR	120	Women and men	40–65	Mean 27	Dyslipidemic	Purified anthocyanins from bilberry and black currant	NA	320 mg/day	Placebo capsule (maltodextrin and pullalan)	12 weeks	Triglycerides, TC, LDL, HDL, SBP, and DBP
Soltani *et al.*, 2014 [22]	Iran	Parallel	54	50	Women and men	≥18	Mean 25	Hyperlipidemic	*Vaccinium arctostaphylos* extract	1000 mg/day	90 mg/day	Placebo capsule (calcium phosphate)	4 weeks	Triglycerides, TC, LDL, and HDL
Zhu *et al.*, 2011 [23]	China	Crossover	150	146	Women and men	40–65	Mean 26	Hypercholesterolemic	Purified anthocyanins from bilberry and black currant	NA	320 mg/day	Placebo capsule	12 weeks	SBP and DBP
Zhu *et al.*, 2013 [24]	China	Parallel	150	146	Women and men	40–65	Mean 26	Hypercholesterolemic	Purified anthocyanins from bilberry and black currant	NA	320 mg/day	Placebo capsule	24 weeks	Triglycerides, TC, LDL, and HDL

^1^ BMI, body mass index; CVD, cardiovascular disease; DBP, diastolic blood pressure; HDL, high-density lipoprotein; LDL, low-density lipoprotein; MetS, metabolic syndrome; MI, myocardial infarction; NA, applicable; NR, not reported; SBP, systolic blood pressure; TC, total cholesterol.

### 2.4. Study Quality Assessment

The 3-category Scottish Intercollegiate Guidelines Network (SIGN) grading system [25] was used to evaluate the overall methodological quality of each article that met the inclusion criteria (Table 1). Each selected article was classified as high quality, acceptable, or unacceptable. An article was graded as high quality if the majority of the outlined criteria were met, there was little or no risk of bias, and the results were unlikely to be changed by further research. An article was graded as acceptable if most of the criteria were met, there were some flaws in the study with an associated risk of bias, and/or the conclusions may change in light of further studies. An article was graded as unacceptable if most of the criteria were not met, there were significant flaws relating to key aspects of the study design, and/or the conclusions were likely to change in light of further studies.

## 3. Results

### 3.1. Study Characteristics

We identified 12 articles [14,15,16,17,18,19,20,21,22,23,24,26] describing 10 studies that assessed the effect of purified anthocyanins or anthocyanin-rich extracts on LDL, HDL, total cholesterol, triglycerides, or blood pressure (Figure 1). One article reported two studies in separate populations in the same publication, one in a healthy population and the other diseased [15]. Others published lipid and blood pressure results from a single trial in separate articles [17,18,23,24]. Finally, another study reported results of high-dose and low-dose anthocyanin administration [16]. Results of these two doses are presented separately in the tables, but they are considered as one study in the tallies in the ensuing discussion. Study characteristics are presented in Table 1.

The overall assessment of risk of bias in each article according to the SIGN criteria was conducted and reported as unacceptable, acceptable, or high quality. Three articles had an overall assessment of high quality [21,22,26], nine articles were of acceptable quality [14,15,16,17,18,19,20,23,24], and no articles were found to have an unacceptable quality rating. Table 2, Table 3 and Table 4 report the overall assessment of risk of bias of each article in proximity to study outcomes, and the full SIGN reviews by criteria are available in Supplementary Table S1.

Of the 10 separate studies, half (*n* = 5) were conducted in Europe [14,16,17,18,19,26]. Two studies were carried out in China [21,23,24], two in Iran [20,22], and one in Mexico [15]. All studies except the study published by Zhu *et al.* [23,24] were single-site trials. All studies except three [15,16,19] reported that they were double-blind in their design.

### 3.2. Lipoproteins

Table 2 shows the results of 10 lipoprotein studies that reported a LDL, HDL, and/or total cholesterol response to anthocyanin supplement interventions. Nine of the 10 studies included LDL as an outcome evaluated for statistical significance in the intervention group compared with the control. Four of these nine studies reported a significant decrease in LDL by the anthocyanin intervention [20,21,22,24]. Notably, only the studies conducted in hyperlipidemic populations demonstrated a decrease in LDL [20,21,22,24]. None of the studies conducted with healthy individuals or other cardiovascular-related disease statuses experienced a significant change in LDL [15,16,19,26].

**Table 2:** Lipoproteins ^1^.

Reference	SIGN Quality	Anthocyanin Dose (mg/Day)	CVD-Related Disease Status	LDL (mg/dL)				HDL (mg/dL)				Total Cholesterol (mg/dL)			
				Percent Change in Intervention	Percent Change in Control	Percent Difference Compared with Control	*P*	Percent Change in Intervention	Percent Change in Control	Percent Difference Compared with Control	*P*	Percent Change in Intervention	Percent Change in Control	Percent Difference Compared with Control	*P*
Curtis *et al.*, 2009 [26]	H	500	Healthy	0.00	−5.71	5.71	NS	0.00	0.00	0.00	NS	1.85	−3.64	5.49	NS
Gurrola-Díaz *et al.*, 2010 [15]	A	19.24	Healthy	−4.12	####	10.73	NR	3.88	0.86	3.03	NR	−4.58	−10.45	5.87	NR
Hansen *et al.*, 2005, full [16]	A	71 or 48	Healthy	0.93	−1.26	2.19	0.643	−6.11	−9.70	3.59	<0.001	−1.48	−5.59	4.10	0.405
Hansen *et al.*, 2005, half [16]	A	36 or 24	Healthy	3.42	−1.26	4.68	0.643	−6.11	−9.70	3.59	<0.001	−1.36	−5.59	4.22	0.405
Karlsen *et al.*, 2007 [19]	A	300	Healthy	—	—	—	—	−1.67	−2.63	0.96	NS	−0.16	−2.38	2.22	NS
Gurrola-Díaz *et al.*, 2010 [15]	A	19.24	MetS	−1.81	2.17	−3.98	NS	30.06	23.47	6.59	0.002	1.10	6.96	−5.86	0.019
Hassellund *et al.*, 2013 [18]	A	640	Prehypertensive	ND	ND	ND	0.341	ND	ND	ND	0.043	ND	ND	ND	0.432
Kianbakht *et al.*, 2014 [20]	A	7.35	Hyperlipidemic	−32.04	−9.12	−22.92	0.002	36.63	2.52	34.11	<0.001	−28.29	−2.76	−25.53	<0.001
Naruszewicz *et al.*, 2007 [14]	A	~64	Post-MI	−0.34	−5.82	5.48	NS	2.84	1.75	1.09	NS	0.91	−3.07	3.98	NS
Qin *et al.*, 2009 [21]	H	320	Dyslipidemic	−12.12	−0.76	−11.37	<0.001	11.55	1.74	9.81	<0.001	−2.52	−0.85	−1.67	0.435
Soltani *et al.*, 2014 [22]	H	90	Hyperlipidemic	−8.61	2.71	−11.32	0.004	−0.35	−1.89	1.54	0.631	−15.21	1.51	−16.71	<0.001
Zhu *et al.*, 2013 [24]	A	320	Hyperlipidemic	−10.42	0.30	−10.72	0.030	12.30	−0.81	13.10	0.036	−4.19	−3.55	−0.64	0.556

^1^ Percent change is calculated as follows: (end of study value − baseline value)/(baseline value) × 100. A, acceptable; CVD, cardiovascular disease; H, high quality; HDL, high-density lipoprotein; LDL, low-density lipoprotein; MetS, metabolic syndrome; MI, myocardial infarction; ND, not determined; NR, not reported; NS, not significant; SIGN, Scottish Intercollegiate Guidelines Network.

Of the 10 lipoprotein studies, all reported the impact of intervention on HDL through statistical comparison against the control. Six studies reported a significant increase in HDL with the anthocyanin intervention [15,16,18,20,21,24]. Three of the six studies showing statistically significant increases in HDL were in subjects with hyperlipidemia [20,21,24]. One study among healthy individuals [16], one study assessing those with metabolic syndrome [19], and one study assessing those with prehypertension [18] also found statistically significant increases in HDL.

Finally, all 10 of the lipoprotein studies reported the impact of intervention on total cholesterol versus the control. Three of the 10 studies reported a significant improvement in the intervention compared with the control [15,20,22]. This includes one study in subjects with metabolic syndrome, in which both the control and intervention groups experienced increases in total cholesterol, but the magnitude of the increase was smaller in the intervention group [15]. By contrast, subjects with hyperlipidemia in the other two studies experienced dramatic lowering of LDL by 16.7% and 25.5%, respectively, in the intervention group compared with the control [20,22]. 

### 3.3. Triglycerides

Table 3 presents the nine studies that assessed triglyceride response to anthocyanin supplement interventions. Of the eight studies that reported a statistical comparison between intervention and control groups, two witnessed a significant reduction in triglycerides [20,22]. Both were conducted in subjects with hyperlipidemia. 

**Table 3:** Triglycerides ^1^.

Reference	SIGN Quality	CVD-Related Disease Status	Anthocyanin Dose (mg/Day)	Triglycerides (mg/dL)			
				Percent Change in Intervention	Percent Change in Control	Percent Difference Compared with Control	*P*
Curtis *et al.*, 2009 [26]	H	Healthy	500	11.11	11.11	0.00	NS
Gurrola-Díaz *et al.*, 2010 [15]	A	Healthy	19.24	−8.24	−19.73	11.50	NR
Gurrola-Díaz *et al.*, 2010 [15]	A	MetS	19.24	−37.94	−17.04	−20.91	NS
Hassellund *et al.*, 2013 [18]	A	Prehypertension	640	ND	ND	ND	0.127
Kianbakht *et al.*, 2014 [20]	A	Hyperlipidemic	7.35	−18.67	−9.68	−8.99	0.002
Naruszewicz *et al.*, 2007 [14]	A	Post-MI	64	−6.15	−3.49	−2.66	NS
Qin *et al.*, 2009 [21]	H	Dyslipidemic	320	−4.24	−2.62	−1.62	0.576
Soltani *et al.*, 2014 [22]	H	Hyperlipidemic	90	−30.79	3.76	−34.55	<0.001
Zhu *et al.*, 2013 [24]	A	Hyperlipidemic	320	−4.08	−2.90	−1.18	0.462

^1^ Percent change is calculated as follows: (end of study value − baseline value)/(baseline value) × 100. A, acceptable; H, high quality; CVD, cardiovascular disease; MetS, metabolic syndrome; MI, myocardial infarction; ND, not determined; NR, not reported; NS, not significant; SIGN, Scottish Intercollegiate Guidelines Network.

### 3.4. Blood Pressure

Table 4 shows the results of the seven studies that reported blood pressure response to anthocyanin supplement interventions. Six of the seven studies conducted a statistical test for systolic and diastolic blood outcomes in response to the intervention versus the control. Only one study that assessed individuals after myocardial infarction showed a significant decrease in systolic and diastolic blood pressure compared with the control [14].

**Table 4:** Blood pressure ^1^.

Reference	SIGN Quality	CVD-Related Disease Status	Anthocyanin Dose (mg/Day)	Systolic Blood Pressure (mmHg)				Diastolic Blood Pressure (mmHg)			
				Percent Change in Intervention	Percent Change in Control	Percent Difference Compared with Control	*P*	Percent Change in Intervention	Percent Change in Control	Percent Difference Compared with Control	*P*
Curtis *et al.*, 2009 [26]	H	Healthy	500	0.81	−4.62	5.43	NR	−1.28	−2.44	1.16	NR
Gurrola-Díaz *et al*., 2010 [15]	A	Healthy	19.2	ND	−2.09	ND	NS	ND	−2.62	ND	NS
Hansen *et al.*, 2005, full [16]	A	Healthy	71 or 48	−4.48	−3.13	−1.35	0.605	−3.66	−5.00	1.34	0.261
Hansen *et al.*, 2005, half [16]	A	Healthy	36 or 24	−1.61	−3.13	1.51	0.605	−1.27	−5.00	3.73	0.261
Gurrola-Díaz *et al.*, 2010 [15]	A	MetS	19.2	−5.57	−9.04	3.47	NS	−11.25	−2.96	−8.29	NS
Hassellund *et al.*, 2012 [17]	A	Prehypertension	640	−5.59	−6.99	1.40	0.254	−13.54	−14.58	1.04	0.324
Naruszewicz *et al.*, 2007 [14]	A	Post-MI	64	−8.32	4.36	−12.68	<0.001	−8.34	1.70	−10.04	<0.001
Qin *et al.*, 2009 [21]	H	Dyslipidemic	320	−0.95	−3.33	2.38	0.888	0.00	−0.97	0.97	0.343
Zhu *et al.*, 2011 [23]	A	Hyperlipidemic	320	−5.31	−0.40	−4.91	0.245	−2.24	−1.93	−0.31	0.290

^1^ Percent change is calculated as follows: (end of study value − baseline value)/(baseline value) × 100. A, acceptable; H, high quality; CVD, cardiovascular disease; MetS, metabolic syndrome; MI, myocardial infarction; ND, not determined; NR, not reported; NS, not significant; SIGN, Scottish Intercollegiate Guidelines Network.

## 4. Discussion

An inverse relationship between anthocyanins and anthocyanin-rich foods and CVD outcomes (e.g., mortality) has been observed among epidemiological studies. McCullough *et al.* recently observed a significant inverse dose-response relationship among 38,180 men and 60,289 women in regard to anthocyanin intake (3.8–22.2 mg/day) and age-adjusted CVD mortality [6]. This systematic review of RCTs suggests that anthocyanins may have potential to influence CVD development and progression among individuals with elevated risk biomarkers. Although most of the potential effects seen in this review were nonsignificant, improvement of biomarkers were consistent across studies, particularly in those with elevated risk biomarkers at baseline. 

CVD development and progression is slow and may span decades. Nutritional interventions often show small changes in the short term but clear effects over the lifespan. Interestingly, trials that used high doses of purified anthocyanins did not observe much more of an effect compared with those studies of anthocyanin-rich extracts containing more physiologically achievable intake. No dose-response relationships were identified among the RCTs included in this review. It is possible that other dietary bioactive components present in the anthocyanin-rich extracts may exert synergistic effects or contribute to a threshold effect. 

Results from animal studies suggest that anthocyanins and other polyphenols may slow or inhibit the absorption of lipids and glucose in the intestine. It has been reported that tea catechins may improve lipid profiles by inhibiting the micelle formation by bile acid [27]. Another possible mechanism for cholesterol-lowering effects of anthocyanins could be the inhibition of cholesterol synthesis. It has been shown that anthocyanins can activate AMP-activated protein kinase (AMPK) [28,29], which is involved in the regulation of energy homeostasis and influences the activity of many enzymes. One enzyme that is inhibited by AMPK is 3-hydroxy-3-methylglutaryl-coenzyme A (HMG-CoA) reductase [30]. Because HMG-CoA reductase is the limiting enzyme of cholesterol synthesis, increased AMPK activity would inhibit cholesterol synthesis and consequently lead to lower cholesterol levels. Furthermore, AMPK inhibits the activity of acetyl-CoA carboxylases ACC1 and ACC2, which leads to increased fatty acid oxidation and decreased fatty acid synthesis [30], and, accordingly, lower triglyceride concentrations. It is possible that anthocyanins may have the ability to modulate low-grade inflammation, as consumption of anthocyanin-rich foods such as berries have been suggested to affect many inflammatory markers of CVD *in vivo* [4]. Berry consumption has been suggested to be an effective strategy to counteract postprandial metabolic and oxidative stress associated with CVD, especially lipid oxidation [31]. Specific berries such as freeze-dried strawberries [32], bilberries and lingonberries [33], blueberries [34] and cranberry extracts [35], among others have shown similar favorable effects on lipid profiles those with elevated markers as suggested in our review.

In regard to blood pressure, anthocyanins have been shown to lower direct measures of arterial stiffness in a cross-sectional study of 1898 women [5]. Across the studies, most did not observe and affect of purified anthocyanins or anthocyanin-rich extracts on blood pressure.

This systematic review identified several gaps in the literature. The age range of participants in most studies was large and may contribute to the null findings of many studies, because it is likely that interventions may have a greater effect among older populations and/or those with an elevated risk of developing CVD. Compliance was also not reported in many of the included studies.

In studies that were not so heterogeneous, a meta-analysis would be carried out to assess the magnitude of effect of purified anthocyanins and anthocyanin-rich extracts on CVD biomarkers. However, we concluded from qualitative review that a meaningful summary estimate could not be obtained by meta-analysis due to high heterogeneity. The control groups included a range of different treatments (e.g., preventative diet). The duration of the studies (3–24 weeks), anthocyanin dose administered (7.35–640 mg/day), composition of the anthocyanin-rich extracts, variation in baseline status of the biomarkers, and difference in the reporting of outcome measures (e.g., mean absolute difference, or percent change) also varied. 

For the purpose of developing recommended intakes, future intervention studies should be designed to assess whether a dose-response relationship of anthocyanins on markers of CVD exists. From this review, we identified gaps in the research literature that could be addressed in further studies. Data in “healthy” individuals (*i.e.*, those with risk biomarkers in the normal range) are important in developing dietary guidance for the general population; however, these data are hard to obtain in short-term clinical studies with a small population. Because larger clinical studies spanning a decade or more are expensive and difficult to control, reliance on well-designed epidemiological studies may be useful in complementing smaller clinical trial data. It may be helpful for future trials to focus on a particular anthocyanin (e.g., Cy-3-glu the most common anthocyanin present in nature), because the type of aglycon and amount of glycosylation and/or acylation of the compounds may significantly alter their biological activity as well as their transportation across the basolateral membrane. By contrast, anthocyanins are currently solely consumed as mixtures in plant-derived foods and extracts. Thus, continued research in this area is equally important. Data on study compliance and evaluation of baseline status of flavonoid and/or polyphenol intakes may improve the consistency between small clinical interventions.

## 5. Conclusions 

Anthocyanins are abundant among plant-derived foods that are currently recommended among national and international dietary guidelines. This systematic review of RCTs adds to existing scientific evidence from observational, animal, and mechanistic studies suggesting that anthocyanins and anthocyanin-rich extracts may have the potential to affect markers of CVD. However, more carefully controlled longer-duration trials assessing dose response across various populations are needed to adequately determine whether an effect of supplementation exists. Current trials suggest that these compounds may decrease LDL cholesterol among individuals with elevated markers, with little to no safety concerns.

## Supplementary Materials

Supplementary materials can be accessed at: http://www.mdpi.com/2072-6643/8/1/32/s1, Table S1: SIGN checklist for controlled trials.
